# Association of Optimism With Cardiovascular Events and All-Cause Mortality: A Systematic Review and Meta-analysis

**DOI:** 10.1001/jamanetworkopen.2019.12200

**Published:** 2019-09-27

**Authors:** Alan Rozanski, Chirag Bavishi, Laura D. Kubzansky, Randy Cohen

**Affiliations:** 1 Department of Cardiology, Mount Sinai St. Luke’s Hospital, New York, New York; 2 Department of Cardiology, Mount Sinai Heart, New York, New York; 3 Department of Cardiology, Icahn School of Medicine at Mount Sinai, New York, New York; 4 Department of Cardiology, Rhode Island Hospital, Warren Alpert Medical School of Brown University, Providence; 5 Department of Social and Behavioral Sciences, Harvard T.H. Chan School of Public Health, Boston, Massachusetts; 6 Department of Cardiology, Crystal Run Healthcare, West Nyack, New York

## Abstract

**Importance:**

Optimism and pessimism can be easily measured and are potentially modifiable mindsets that may be associated with cardiovascular risk and all-cause mortality.

**Objective:**

To conduct a meta-analysis and systematic review of the association between optimism and risk for future cardiovascular events and all-cause mortality.

**Data Sources and Study Selection:**

PubMed, Scopus, and PsycINFO electronic databases were systematically searched from inception through July 2, 2019, to identify all cohort studies investigating the association between optimism and pessimism and cardiovascular events and/or all-cause mortality by using the following Medical Subject Heading terms: *optimism*, *optimistic explanatory style*, *pessimism*, *outcomes*, *endpoint*, *mortality*, *death*, *cardiovascular events*, *stroke*, *coronary artery disease*, *coronary heart disease*, *ischemic heart disease,* and *cardiovascular disease*.

**Data Extraction and Synthesis:**

Data were screened and extracted independently by 2 investigators (A.R. and C.B.). Adjusted effect estimates were used, and pooled analysis was performed using the Hartung-Knapp-Sidik-Jonkman random-effects model. Sensitivity and subgroup analyses were performed to assess the robustness of the findings. The Meta-analysis of Observational Studies in Epidemiology (MOOSE) reporting guideline was followed.

**Main Outcomes and Measures:**

Cardiovascular events included a composite of fatal cardiovascular mortality, nonfatal myocardial infarction, stroke, and/or new-onset angina. All-cause mortality was assessed as a separate outcome.

**Results:**

The search yielded 15 studies comprising 229 391 participants of which 10 studies reported data on cardiovascular events and 9 studies reported data on all-cause mortality. The mean follow-up period was 13.8 years (range, 2-40 years). On pooled analysis, optimism was significantly associated with a decreased risk of cardiovascular events (relative risk, 0.65; 95% CI, 0.51-0.78; *P* < .001), with high heterogeneity in the analysis (*I*^2^ = 87.4%). Similarly, optimism was significantly associated with a lower risk of all-cause mortality (relative risk, 0.86; 95% CI, 0.80-0.92; *P* < .001), with moderate heterogeneity (*I*^2^ = 73.2%). Subgroup analyses by methods for assessment, follow-up duration, sex, and adjustment for depression and other potential confounders yielded similar results.

**Conclusions and Relevance:**

The findings suggest that optimism is associated with a lower risk of cardiovascular events and all-cause mortality. Future studies should seek to better define the biobehavioral mechanisms underlying this association and evaluate the potential benefit of interventions designed to promote optimism or reduce pessimism.

## Key Points

**Question:**

Is a mindset of optimism associated with a lower risk of cardiovascular events and all-cause mortality?

**Findings:**

In this meta-analysis of 15 studies including 229 391 individuals, optimism was associated with a lower risk of cardiovascular events and pessimism was associated with a higher risk of cardiovascular events; the pooled association was similar to that of other well-established cardiac risk factors.

**Meaning:**

The findings suggest that a mindset of optimism is associated with lower cardiovascular risk and that promotion of optimism and reduction in pessimism may be important for preventive health.

## Teaser

This meta-analysis assesses whether individuals with a mindset of optimism have a lower risk of cardiovascular events and all-cause mortality.

## Introduction

Extensive evidence has demonstrated an association between negative emotions, social factors, and certain chronic stress conditions and adverse cardiac outcomes.^1^ Less well studied has been the potential association between positive and negative mindsets and cardiac risk. Such research is of interest because mind-sets are potentially modifiable, thus making them a novel relevant target for clinical intervention. One such mindset is an individual’s level of optimism, commonly defined as the tendency to think that good things will happen in the future.^2^ Empirical studies have long indicated that more optimistic individuals are more likely to succeed at work and in school, sports, politics, relationships, and other forms of life endeavors.^3,4^ A more recent study also reported positive associations between optimism and a range of favorable physical health outcomes.^5^ Nevertheless, the assessment of optimism and pessimism in cardiac medical practice is uncommon. In 2001, Kubzansky and colleagues^6^ reported the first study, to our knowledge, to find an association between higher optimism and a lower risk for specific cardiac outcomes, including angina, myocardial infarction, and cardiac death. They showed effects of optimism beyond those of depression or other forms of psychological distress, a critical finding because a concern about such findings is that they simply reflect the absence of depression rather than active effects of optimism. Since then, similar findings have been described in other studies,^7,8,9,10,11,12,13,14,15,16,17,18,19,20^ and most studies considered depression or distress as a potential confounder. To consider these findings more systematically, we conducted a meta-analysis of studies that have assessed the association between optimism and pessimism and adverse cardiac outcomes. Our goals were to evaluate the magnitude of this association, the consistency of results among reported studies, the influence of potential confounders, and the quality of the reported literature.

## Methods

### Data Sources and Searches

For this systematic review and meta-analysis, PubMed, Scopus, and PsycINFO databases were systematically searched for articles published from inception through July 2, 2019, with the following Medical Subject Heading terms: *optimism*, *optimistic explanatory style*, *pessimism*, *outcomes*, *endpoint*, *mortality*, *death*, *cardiovascular events*, *stroke*, *coronary artery disease*, *coronary heart disease*, *ischemic heart disease,* and *cardiovascular disease*. No language restrictions were imposed for the search. In addition, references from included studies and pertinent review articles were searched to identify other studies meeting selection criteria. The present systematic review and meta-analysis was conducted and reported according to the recommendations of the Meta-analysis of Observational Studies in Epidemiology (MOOSE) reporting guideline.^21^

### Study Selection

Two of us (A.R. and C.B.) independently identified articles eligible for review. Articles were selected for inclusion in the meta-analysis if the study evaluated associations of optimism with all-cause mortality and/or cardiovascular events and reported adjusted risk estimates with 95% CIs. Studies of patients with cancer were excluded to avoid confounding secondary to terminal sickness. Articles were identified for further review by performing an initial screen of abstracts, followed by full-text reviews. Only empirical articles were considered. With regard to multiple studies from the same data set, only 1 article was included and was selected based on relevance, clearly defined outcomes, and larger sample size.

### Data Extraction and Quality Assessment

Data were independently extracted by 2 of us (A.R. and C.B.) using a standardized protocol and reporting form. Disagreements were resolved by arbitration, and consensus was reached after discussion. The following information was extracted: study characteristics (study name, authors, publication year, country of origin, sample size, study design, and follow-up duration), study sample characteristics (mean age, sex, and major comorbidities), main exposure (method of assessment of optimism or pessimism) and main outcomes (all-cause mortality and/or cardiovascular events). Adjusted relative risks (RRs) or hazard ratios, 95% CIs, and information about the variables used for adjustment in multivariable analysis were abstracted. Study quality was assessed by the Newcastle-Ottawa scale,^22^ with quality grades assigned based on selection of the study groups, comparability, and assessment of outcomes.

### Statistical Analysis

For the present analysis, only adjusted RRs or hazards ratios and 95% CIs reported by individual studies were used, which reflect estimates with the most complete adjustment available for baseline covariates. Because of known clinical and methodologic heterogeneity of studies, effect estimates were pooled using Hartung-Knapp-Sidik-Jonkman random-effects models.^23^ When studies reported separate RRs for men and women from the same cohort, data were included from both men and women separately in the pooled analysis. Most studies compared multiple categories of optimism (often categorized according to tertiles or quartiles based on score distribution in a particular sample), reporting effect estimates for highest levels of optimism vs lowest reference categories. When effect estimates for optimism and outcomes were reported according to categorical optimism levels and also using optimism as a continuous variable, effect estimates were selected for the highest categorical level of optimism. Studies by Brummett et al,^7^ Grossardt et al,^8^ and Mosing et al^9^ reported effect estimates for pessimism and all-cause mortality, for which we used reciprocal values of RR or hazards ratio to ensure uniform statistical analysis.

All-cause mortality and cardiovascular events were analyzed as separate outcomes. Cardiovascular events predominantly included cardiovascular or coronary heart disease mortality; in 2 studies, the occurrence of nonfatal myocardial infarction and/or new onset angina were also included as cardiovascular events.^6,10^ Heterogeneity among studies was assessed using Higgins and Thompson *I*^2^ statistics. The *I*^2^ is the proportion of total variation observed among the studies that is attributable to differences between studies rather than sampling error (chance), with *I*^2^ values corresponding to the following levels of heterogeneity: low (<25%), moderate (25%-75%), and high (>75%).^24^ Reasons for heterogeneity in study results were further explored using subgroup analyses. Subgroup analyses were performed according to assessment method for optimism or pessimism, follow-up duration, sex, geographical location, and determination of whether studies assessed or did not assess the effects of critical potential confounders, including depression, educational level, and health behavior as measured by physical activity. Other health behaviors were not considered because too few studies reported on them. In addition, there were sufficient data to assess whether findings differed with and without consideration of educational level as a separate covariate. We also performed a sensitivity analysis to investigate the association of each individual study with the overall meta-analysis results. Publication bias was tested using the Begg correlation test^25^ and visual inspection of a funnel plot. Publication bias tests could be highly limited because of a smaller number of studies.^26^ The Duval and Tweedie nonparametric trim-and-fill procedure was used to further assess the possible effect of publication bias in our meta-analysis.^27^ The Duval and Tweedie trim-and-fill method uses an iterative procedure (1000 iterations used in this study) to remove (ie, trim) smaller studies that cause funnel plot asymmetry and thus publication bias, uses the trimmed funnel plot to estimate the true center of the funnel plot, and then replaces the omitted studies and their missing counterparts around the center (ie, fill). A 2-tailed *P* < .05 was considered to be statistically significant. All analyses were performed using Stata statistical software, version 16 (StataCorp).

## Results

### Study Characteristics

**Figure 1.**
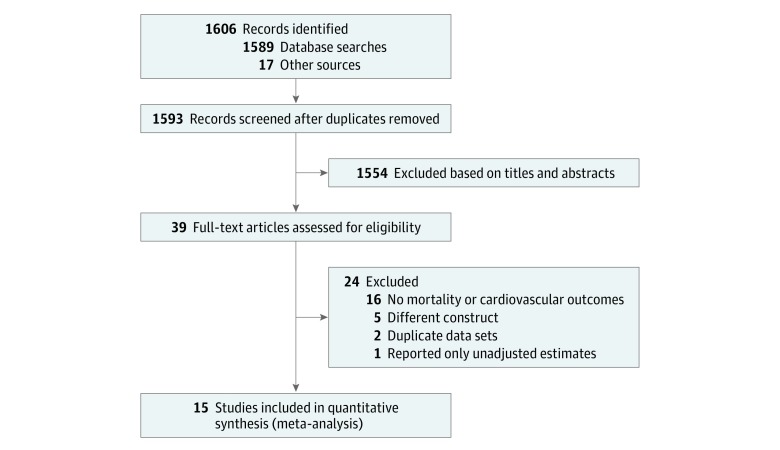
Flowchart of Study Selection

A flow diagram of the literature search and related screening process is shown in Figure 1. A total of 15 studies^6,7,8,9,10,11,12,13,14,15,16,17,18,19,20^ published between November 2001 and January 2017 met our inclusion criteria. Of these, 14 were prospective studies and 1 was a retrospective cohort study.^8^ Overall characteristics of the included studies, which comprised 229 391 individuals, are summarized in Table 1.

**Table 1.** Characteristics of Studies Included in the Meta-analysis

Source	Group	Study Period	Participants, No.	Male, %	Mean Age (Range), y	Follow-up, y	Assessment of Optimism	End Point
Anthony et al,^11^ 2016	Community cohort	1999-2002	876	41.9	74.1 (30-79)	8.1	LOT-R	ACM and CVD mortality
Boehm et al,^10^ 2011	Community cohort	1991-1994	7942	69.1	49.5 (39-63)	5.4	Single-item questionnaire	CHD mortality, nonfatal MI, and new angina
Brummett et al,^7^ 2006	University students	1964-1966	5750	82.6	18.5 (NR)	40.0	MMPI subscale	ACM
Engberg et al,^12^ 2013	Nonagenerians	1998	2262	25.8	NR (92-93)	12.0	1-Item questionnaire	ACM
Giltay et al,^13^ 2004	Elderly individuals	1991	941 (494)^a^	49.5	74.5 (65-85)	9.1	7-Item questionnaire	ACM and CVD mortality
Giltay et al,^14^ 2006	Elderly Individuals	1985-1990	545	100	71.7 (64-84)	15.0	4-Item questionnaire	CVD mortality
Grossardt et al,^8^ 2009	Ambulatory patients	1962-1965	7080	48.7	48.1 (38-57)	32.4	MMPI subscale	ACM
Hansen et al,^15^ 2010	Community cohort	1995	1739	49.6	46.2 (NR)	10.0	2 Items from LOT-R	CHD mortality
Kim et al,^16^ 2011	Community cohort	2006-2008	6044	42	68.5 (>50)	2.0	LOT-R	Stroke
Kim et al,^17^ 2017	Female nurses	2004-2014	70 021	0	70.1 (36-55)	9.0	LOT-R	ACM and CVD mortality
Kubzansky et al,^6^ 2001	Community cohort	1986	1306	100	60.8 (21-80)	10.0	MMPI subscale	CHD mortality and nonfatal MI
Mosing et al,^9^ 2012	Twin participants, >50 y	1993-1995	3752	31	61.3 (>50)	16.0	LOT-R	ACM
Nabi et al,^18^ 2010	Community cohort	1998-2005	23 216	41	NR (20-54)	7.0	LOT-R	Stroke
Tindle et al,^19^ 2009	Postmenopausal women cohort	1994-1998	97 253	0	NR (50-79)	8.0	LOT-R	ACM and CVD mortality
Weiss-Faratci et al,^20^ 2017	Patients after MI	1992-1993	664	85.2	52.4 (<65)	22.4	LOT-R	ACM

Abbreviations: ACM, all-cause mortality; CHD, coronary heart disease; CVD, cardiovascular disease; LOT-R, Life Orientation Test–Revised scale; MI, myocardial infarction; MMPI, Minnesota Multiphasic Personality Inventory; NR, not recorded.

^a^ Subgroup of patients who reported cardiovascular events.

Of the 15 studies, 8 were conducted in the United States,^6,7,8,11,15,16,17,19^ 5 in Europe,^10,12,13,14,18^ 1 in Israel,^20^ and 1 in Australia.^9^ Ten studies reported data on cardiovascular events,^6,10,11,13,14,15,16,17,18,19^ and 9 studies reported data on all-cause mortality.^7,8,9,11,12,13,17,19,20^ The mean follow-up period was 13.8 years (range, 2-40 years). With the exceptions of a study that assessed ambulatory patients^8^ and another that assessed patients who had previous myocardial infarction,^20^ all studies were performed in community cohorts of different ages. Mean age at baseline assessment ranged from 19 to 93 years. Details regarding how exposures and outcomes were assessed in the included studies are shown in eTable 1 in the Supplement. A variety of scales were used to assess optimism and pessimism. The most frequently used (in 8 of 15 studies) scale was the Life Orientation Test–Revised.^28^ All studies had a low risk for bias per the Newcastle-Ottawa scale (eTable 2 in the Supplement).

### Optimism and Incident Cardiovascular Events

**Figure 2.**
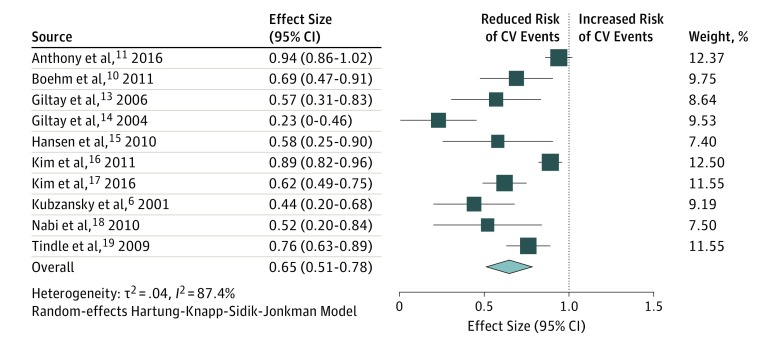
Association Between Optimism and Cardiovascular (CV) Events Boxes indicate mean values, with larger boxes indicating greater weight; whiskers represent 95% CIs; and the diamond indicates the pooled mean value with the tips of the diamond representing the 95% CI of the pooled mean.

The 10 studies reporting on cardiovascular events included 209 436 participants. On pooled analysis, optimism was significantly associated with reduced risk of cardiovascular events (RR, 0.65; 95% CI, 0.51-0.78; *P* < .001) (Figure 2). A high heterogeneity was observed in the analysis (*I*^2^ = 87.4%). Exclusion of the study by Tindle et al,^19^ the largest study, did not result in any change in the pooled result (RR, 0.63; 95% CI, 0.48-0.78; *P* = .001). Visual inspection of the funnel plot showed evidence of publication bias (smaller studies showing no beneficial effects were missing). According to the trim-and-fill method, the association between optimism and cardiovascular events remained significant after imputing 4 possible missing studies (adjusted RR, 0.77; 95% CI, 0.61-0.92; *P* < .001) (eFigure in the Supplement).

### Optimism and All-Cause Mortality

**Figure 3.**
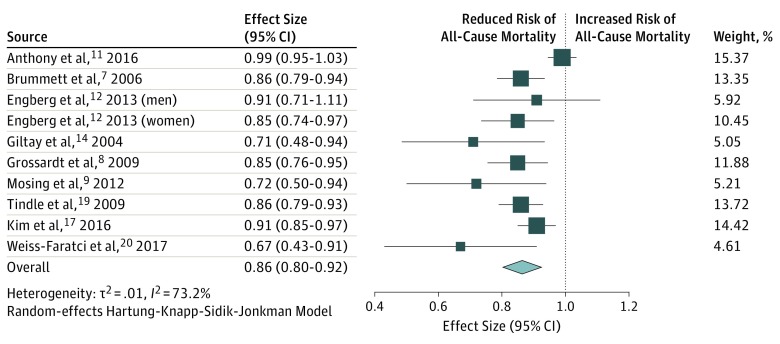
Association Between Optimism and All-Cause Mortality Boxes indicate mean values, with larger boxes indicating greater weight; whiskers represent 95% CIs; and the diamond indicates the pooled mean value with the tips of the diamond representing the 95% CI of the pooled mean.

The 9 studies (10 comparisons) reporting on all-cause mortality included 188 599 participants. On pooled analysis, optimism was significantly associated with reduced risk of all-cause mortality (RR, 0.86; 95% CI, 0.80-0.92; *P* < .001) (Figure 3). Moderate heterogeneity was observed in the analysis (*I*^2^ = 73.2%). Exclusion of the study by Tindle et al^19^ did not result in any change in the pooled result (RR, 0.86; 95% CI, 0.79-0.93; *P* < .001). Visual inspection of a funnel plot showed evidence of publication bias. According to the trim-and-fill method, the association between optimism and all-cause mortality remained significant after imputing 4 possible missing studies (adjusted RR, 0.90; 95% CI, 0.83-0.97; *P* < .001) (eFigure in the Supplement).

### Subgroup Analysis

Subgroup analysis by method of assessment for optimism, follow-up duration, sex, study location, depression, educational level, socioeconomic status, and exercise or physical activity yielded largely similar results for the associations between optimism and pessimism and the risk for either cardiovascular events or all-cause mortality (Table 2).

**Table 2.** Relative Risk of Adverse Events Associated With Optimism Within Subgroups

Measures	Pooled Relative Risk (95% CI)	
	Cardiovascular Events	All-Cause Mortality
Measurement scale		
Life Orientation Test–Revised	0.71 (0.57-0.86)	0.87 (0.78-0.96)
Other	0.50 (0.23-0.77)	0.84 (0.76-0.93)
Predominant sex cohort^a^		
Male	0.57 (0.41-0.74)	0.81 (0.70-0.93)
Female	0.67 (0.49-0.85)	0.89 (0.82-0.95)
Country		
United States	0.73 (0.60-0.86)	0.90 (0.85-0.96)
Other	0.42 (0.20-0.65)	0.79 (0.69-0.90)
Depression		
Adjusted	0.66 (0.54-0.77)	0.85 (0.73-0.97)
Not adjusted	0.64 (0.43-0.86)	0.87 (0.80-0.94)
Follow-up, y		
<10	0.68 (0.51-0.86)	0.90 (0.79-1.00)
≥10	0.52 (0.36-0.68)	0.83 (0.76-0.91)
Educational level		
Adjusted	0.60 (0.43-0.76)	0.84 (0.76-0.93)
Not adjusted	0.78 (0.57-0.99)	0.89 (0.80-0.97)
Employment grade or socioeconomic status		
Adjusted	0.69 (0.48-0.91)	0.81 (0.69-0.92)
Not adjusted	0.64 (0.49-0.79)	0.89 (0.82-0.95)
Exercise or physical activity		
Adjusted	0.73 (0.62-0.85)	0.90 (0.80-0.96)
Not adjusted	0.56 (0.32-0.80)	0.83 (0.76-0.91)

^a^ Considered to be predominant if the sex represented more than 50% of the study population.

### Assessment of Linear Trend

Among the 15 studies, optimism and pessimism were assessed solely as a continuous variable in 2 studies.^7,9,11^ In the other 13 studies, participants were divided into either tertiles or quartiles and a statistical assessment was performed regarding the presence or absence of a significant linear trend between levels of optimism and reduced risk for cardiac events and/or all-cause mortality (eTable 1 in the Supplement). In 12 of 15 studies, a significant linear trend was observed.

## Discussion

A review of the literature has noted associations between a number of psychosocial risk factors, including negative emotions such as depression and anxiety, social factors (eg, loneliness), and certain chronic stress conditions, with cardiovascular disease. Specific mindsets, habitual patterns of thinking which influence individuals’ views and interactions, have also been associated with cardiovascular disease risk. Using the strongest epidemiologic methods available, a growing body of research has investigated whether the mindset of optimism vs pessimism might be associated with cardiovascular disease and has also explored potential mechanisms underlying these associations. Herein, we report the results of a comprehensive systematic review and meta-analysis to assess the association between optimism and pessimism and adverse cardiovascular outcomes.

This meta-analysis consisted of 15 studies^6,7,8,9,10,11,12,13,14,15,16,17,18,19,20^ involving 229 391 participants. Ten of the studies assessed the association between optimism and pessimism and adverse cardiovascular outcomes. In 9 of 10 studies,^6,10,11,13,14,15,16,17,18,19^ there was a significantly lower risk of cardiovascular events among individuals with high optimism scores after adjustment for a variety of clinical measures in each study. The overall pooled risk ratio for cardiovascular events among individuals with high optimism levels was 0.65. Among 9 studies,^7,8,9,11,12,13,17,19,20^ optimism was also associated with a reduction in all-cause mortality, but the decrease in risk was more modest, with an overall pooled risk ratio of 0.86. As with cardiovascular events, the results among studies were consistent, with 8 of 9 studies showing lower risk of all-cause mortality among the most optimistic individuals.

### Methodologic Differences Among Studies

There was considerable variation in questionnaires used to assess optimism and pessimism. Most studies queried dispositional optimism, with the Life Orientation Test–Revised^28^ most commonly used (in 8 of 15 studies). Three studies^6,7,8^ used an explanatory style measure of optimism derived using items from the Minnesota Multiphasic Personality Inventory,whereas 2 studies^10,12^ used a single-item measure. Despite this heterogeneity in how optimism was assessed, the lower RR that was associated with optimism was comparable among studies. Studies also varied by length of follow-up. Among the 15 studies, participants were followed for at least 10 years in 8 studies.^6,7,8,9,12,14,15,20^ A significantly lower risk of cardiovascular events and all-cause mortality was observed across studies regardless of follow-up duration.

### Assessment of Potentially Confounding Variables

In general, the risk ratios used for this meta-analysis were adjusted for a variety of potentially confounding clinical variables. Most studies adjusted for some if not all major cardiac disease risk factors. Many studies also adjusted for psychological distress to rule out concerns that associations were primarily attributable to the absence of depression, and approximately half of the studies presented estimates also adjusted for educational level and physical activity. Protective effects of optimism were maintained among studies adjusting for these variables. In addition, optimism was associated with comparably reduced risk among studies with a predominance of men vs women and among studies conducted in the United States vs other countries.

### Assessment of Outcomes According to the Magnitude of Optimism and Pessimism

Optimism was generally assessed according to continuous scales that used multi-item measures, with associations generally estimated according to tertiles or quartiles of optimism. In the 2 studies assessing optimism and pessimism by a single question, participants were divided into 3 categories based on their responses, with comparisons made between the highest vs lowest categories. Evidence of a dose-response association between level of optimism and decreased clinical risk was present in 12 of 15 studies. In 2 of the 3 studies without evidence of a dose-response association,^10,15^ the optimism assessment was limited with measures including only 1 or 2 items. The evidence of dose-response associations paralleled similar findings reported for the clinical hazard of cardiovascular outcomes associated with depression, poor social support, and other psychosocial risk factors.^1^

### Comparison With Studies of Optimism and Other Medical Conditions

Our study was the first meta-analysis, to our knowledge, to assess the association between optimism and clinical outcomes. Findings were consistent with studies^17,29,30,31^ that have evaluated the association between optimism and other related medical conditions. This includes studies that have shown an association between optimism and the risk of heart failure,^29^ development of cognitive dysfunction among elderly persons,^30^ rate of atherosclerotic progression,^31^ and respiratory disease, infection, and various cancers.^17^ In addition, a previous meta-analysis found consistent associations between optimism and a reduced likelihood of various other adverse physical health outcomes.^5^ Combined, these data support the findings of our meta-analysis.

### Mechanisms

Psychosocial risk factors tend to exert their adverse effects by both indirect behavioral mechanisms and direct physiologic mechanisms.^32^ Accumulating data suggest that similar mechanisms may be associated with the presence of optimism and pessimism. With respect to behavioral mechanisms, Boehm and colleagues^33^ conducted a random-effects meta-analysis to examine the association between optimism and 3 cardiac-relevant health behaviors: physical activity, diet, and cigarette smoking. The study^33^ found a positive association between optimism and better health behaviors, although most evidence was cross-sectional and effect sizes were modest. More recently, however, larger studies^34,35,36,37^ with prospective designs have found significant associations between measurement of initial optimism and pessimism levels and subsequent health behaviors. For instance, among participants in the Women’s Health Initiative, greater optimism was associated with both better diet quality^34^ and a greater likelihood of sustaining physical exercise over time.^35^

Studies have also reported associations between optimism and pessimism and a variety of pathophysiologic mediators of chronic disease, including increased inflammation and impairments in hemostasis and endothelial function^38,39^; metabolic function^40^; telomerase activity and telomere length^41,42,43^; ambulatory blood pressure^44^; and hypothalamic-pituitary-adrenocortical function.^45,46,47^ Together, these findings suggest a direct association of optimism vs pessimism with physiologic functioning, although study of individual mediators remains sparse compared with the study of more established psychosocial mediators, such as depression. Prospective study is needed to evaluate whether pessimism is a stronger contributor to pathophysiologic dysfunction (as suggested by some studies^38,40,43^) than optimism is for providing positive physiologic buffering.

### Clinical Significance

Optimism has long been promulgated as a positive attribute for living. The findings of the current meta-analysis suggest that optimism is associated with cardiovascular benefits and that pessimism is associated with cardiovascular risk, with a pooled association that was similar to that for well-established cardiac risk factors. Taken together, the cardiovascular and psychological benefits of optimism make it an attractive new arena for study within the field of behavioral cardiology. The success of this research may require addressing 3 key issues. First, there is a need to define more clearly the central processes that underlie the medical benefits associated with optimism. This should include more study into the physiological processes and health behaviors that may ensue from optimism vs pessimism as well as the study of potential salutogenic mechanisms that may cotravel with optimism vs pessimism. With respect to the latter, a study^48^ reported an association of optimism with more effective goal setting, problem solving, and coping skills, suggesting that these are potential assets related to optimism that could be incorporated into the measurement of optimism and/or form the basis of clinical intervention.

Second, the studies of our meta-analysis were associated with substantial variability in the cut points that were applied to optimism vs pessimism. This variability differs from the use of depression scales, whereby specific diagnostic cut points have been established. For instance, screening for depression, as advocated by the American Heart Association, has been made possible because of the development of widely accepted criteria for defining depression risk based on the 2- and 9-item General Health Questionnaire. A similar consensus in diagnostic criteria could improve future epidemiologic investigations regarding optimism and pessimism and is needed for use as a clinical assessment tool in medical practice. To this end, emerging data suggest that the Life Orientation Test–Revised, as developed by Scheier and colleagues,^28^ may be a suitable screening tool given its brevity and successful use across many clinical outcomes.

Third, the findings of this meta-analysis appear to support establishment of interventions that might diminish pessimism and promote optimism among clinical patients. Various studies^49,50,51,52,53,54^ have reported that pessimism can be reduced^49,50^ and optimism can be enhanced through the use of cognitive behavioral therapy and positive psychological techniques,^51,52,53,54^ making these techniques potentially suitable for use in cardiac rehabilitation programs and other group settings.^55^ However, further research will need to assess whether optimism that is enhanced or induced through directed prevention or intervention strategies has similar health benefits vs optimism that is naturally occurring. More broadly, the present findings concerning the cardiac benefits of optimism might encourage studies on whether similar benefits can be derived from instilling other positive mindsets (eg, sense of purpose or gratitude) that may be elicited through guided interventions.

### Limitations

Our meta-analysis has several limitations. The cohorts included in this meta-analysis varied widely in age, ranging from teenagers in 1 study^7^ to nonagenarians in another.^12^ However, the consistent association of optimism to reduced cardiovascular risk among all age groups could also be considered a strength of our study. Although each study adjusted for important covariates, these covariates varied widely from study to study. Thus, we could not systematically assess the independent effects of various individual clinical covariates, including smoking, diabetes, and hypertension. This variability in covariate adjustment may help to account for the considerable heterogeneity found among studies, which was high for the assessment of cardiovascular outcomes (*I*^2^ = 87.4%) and moderate for all-cause mortality (*I*^2^ = 73.2%). Lack of uniformity in scales and/or variance in the cut points used within a given scale, particularly the Life Orientation Test–Revised, may have also contributed to this heterogeneity. In addition, the measurement of optimism according to both positively and negatively framed items has led to an ongoing debate as to whether these items separately represent optimism and pessimism as distinct constructs. Although recent research suggests that considering both positively and negatively worded items as indicative of a unitary measure of optimism is most appropriate,^56^ there was insufficient information to address this issue in our meta-analysis.

## Conclusions

The findings suggest that optimism is associated with a lower risk of cardiovascular events and all-cause mortality. Future studies should seek to better define the biobehavioral mechanisms underlying this association and evaluate the potential benefit of interventions designed to promote optimism or reduce pessimism.

## Notes

Supplement. **eTable 1.** Exposure and Outcomes Assessments and Adjusted Covariates in the Included Studies **eTable 2.** Quality of Included Studies per Newcastle-Ottawa Scale for Quality Assessment of Cohort Studies **eFigure.** Funnel Plots to Assess Publication Bias for (A) Cardiovascular Events and (B) All-Cause Mortality
