# Supplementary material for: Association of Optimism With Cardiovascular Events and All-Cause Mortality: A Systematic Review and Meta-analysis
Source: JAMA Netw Open. 2019 Sep 27;2(9):e1912200. doi: 10.1001/jamanetworkopen.2019.12200 (PMC6777240; doi:10.1001/jamanetworkopen.2019.12200)
Supplement: Supplement. — eTable 1. Exposure and Outcomes Assessments and Adjusted Covariates in the Included Studies eTable 2. Quality of Included Studies per Newcastle-Ottawa Scale for Quality Assessment of Cohort Studies eFigure. Funnel Plots to Assess Publication Bias for (A) Cardiovascular Events and (B) All-Cause Mortality [file jamanetwopen-2-e1912200-s001.pdf]

Supplementary Online Content

Rozanski A, Bavishi C, Kubzansky LD, Cohen R. Association of optimism with cardiovascular events and all-cause mortality: a systematic review and meta-analysis. *JAMA Netw Open*. 2019;2(9):e1912200. doi:10.1001/jamanetworkopen.2019.12200

eTable 1. Exposure and Outcomes Assessments and Adjusted Covariates in the Included Studies

eTable 2. Quality of Included Studies per Newcastle-Ottawa Scale for Quality Assessment of Cohort Studies

eFigure. Funnel Plots to Assess Publication Bias for (A) Cardiovascular Events and (B) All-Cause Mortality

This supplementary material has been provided by the authors to give readers additional information about their work.

© 2019 Rozanski A et al. *JAMA Network Open.*

eTable 1. Exposure and Outcomes Assessments and Adjusted Covariates in the Included Studies

Study	Categories of Optimism	Outcome assessment	Evidence of linear trend	Adjusted covariates
Anthony et al, 2016	LOT-R score, continuous variable	ICD 9 codes for all-cause and CV mortality	Yes	Age, gender, angina meds, cholesterol-lowering meds, diabetic meds, SF-12 variables including mental component average week alcohol use, smoking status, waist-to-hip ratio, exercise status, score.
Boehm et al, 2011	One question, three categories:<br>Cat 1 Low (reference)<br>Cat 2 Moderate<br>Cat 3 High	National health registry and ICD 10 codes for CV mortality	No	Age, gender, systolic and diastolic blood pressure, high-density cholesterol, low-density cholesterol, triglycerides, body mass index, and diabetes;ethnicity, marital status, grade of employment, smoking status, psychological ill-being alcohol consumption, exercise, fruit and vegetable consumption.
Brummett et al , 2006	MMPI derived scale, continuous variable	National death index, and social security death index for all-cause mortality	Yes	Gender.
Engberg et al, 2014	One question, three categories:<br>Cat 1 Optimistic<br>Cat 2 Neutral (ref)<br>Cat 3 Pessimistic	Danish Civil registration system for all-cause mortality	Yes	Circulatory diseases, cancer (except for skin cancer), diabetes, non-fatal chronic diseases, and lung disease, cognitive function, activities of daily living.
Giltay et al, 2004	Subscale of Dutch Scale of Subjective Well-being:<br>Cat 1 0-8.6 (reference)<br>Cat 2 10-12.9<br>Cat 3 14.3-15.7<br>Cat 4 17.1-20	Municipal registries and ICD 10 codes for all-cause and CV mortality	Yes	Age, smoking (current, former, or never), alcohol education, socioeconomic status, marital status, and total activity score.
Giltay et al, 2006	4 question scale:<br>Cat 10-1.25 (ref)<br>Cat 2 1.33-1.75<br>Cat 3 2.0	Municipal registries and ICD 9 codes for CV mortality	Yes	Age, smoking status, diabetes mellitus, mean arterial pressure, antihypertensive medication, total and high-density lipoprotein cholesterol levels, family history of stroke or myocardial infarction, living arrangement, education, self-rated health, body mass index, physical activity, alcohol use and smoking status.
Grossardt et al, 2009	MMPI derived scale:<br>Cat 1 NR (reference)<br>Cat 2 NR<br>Cat 3NR<br>Cat 4 NR	National death index for all-cause mortality	Yes	Education, self assessed general health, and alcohol use.
Hansen et al, 2010	Abbreviated LOT-R:	ICD 9 and ICD 10 codes for	No	Age, gender, Framingham cardiovascular risk factors, negative and positive affect

© 2019 Rozanski A et al. *JAMA Network Open.*

	Cat 1 High Optimism<br>Cat 2 Moderate<br>Cat 3 Low Optimism (reference)	ischemic heart disease		
Kim et al, 2011	LOT-R optimism subscale, continuous variable	Self-report of physician's diagnosis or proxy reports for patients who had died	Yes	Age, gender, chronic illness, self-reported health, race/ethnicity, marital status, educational degree, current smoker, exercise, alcohol use, diabetes, body mass index, systolic/diastolic blood pressure, hypertension, heart disease
Kim et al, 2016	LOT-R score:<br>Cat 1 Median score 13 (reference)<br>Cat 2 Median score 19<br>Cat 3 Median score 22<br>Cat 4 Median score 24	National death index, supplemented by reports from family members and postal authorities for all-cause mortality	Yes	Age, race, marital status, educational level, husband's educational level, father's occupation when the participant was 16 years of age, depression, high cholesterol, hypertension, type 2 diabetes mellitus, myocardial infarction, stroke, cancer, body mass index, smoking status, physical activity level, alcohol consumption, physical examination for screening purposes, and diet (Alternative Healthy Eating Index).
Kubzansky, et al 2001	MMPI derived scale:<br>Cat 1 15 -41<br>Cat 2 41.1-49.9<br>Cat 3 50-79.7 (reference)	Adjudication of hospital medical records, ICD 8 codes for Cardiac mortality	Yes	Age, family history of CHD (yes or no), educational attainment, systolic and diastolic blood pressure (in mm Hg), serum total cholesterol level (mg/dl), anxiety, depression, anger. And alcohol intake (≥2 drinks per day), BMI (kg/m*2*), smoking status (never, former, or current).
Mosing et al, 2012	LOT-R score, continuous variable	Australian National Death Index, ICD 10 codes for all-cause mortality	Yes	Age, sex, socioeconomic status, smoking, drinking, obese, and exercise.
Nabi et al, 2010	LOT-R score, quartiles of pessimism score	National Hospital Discharge Register and the Statistics Finland Mortality Register	Yes	Age, sex, education, marital status, behavior related risk factors, hypertension, diabetes, depression, general feeling of stressfulness, incident CHD
Tindle et al, 2009	LOT-R score:<br>Cat 1 ≥26<br>Cat 2 24-25<br>Cat 3 22-23<br>Cat 4 <22 (reference)	National death index for all-cause and CV mortality	Yes	Age, race/ethnicity, education, income, observational study cohort vs clinical trial status, diabetes mellitus, hypertension, high cholesterol, HRT use, depressive symptoms, alcohol consumption, smoking, physical activity, and body mass index
Weiss-Faratci et al, 2017	LOT-R score:<br>Cat 1≤ 15 (reference)<br>Cat 2 16-18<br>Cat 3 >18	Israeli Population Registry, medical records, death	No	Age, sex, education, employment status, and partner status, Charlson comorbidity index, Killip class, hypertension, diabetes mellitus, dyslipidemia, depression, social support, smoking and obesity

© 2019 Rozanski A et al. *JAMA Network Open.*

		certificates, family physicians, and family members for all-cause mortality		

Cat = category; NR = not reported.

© 2019 Rozanski A et al. *JAMA Network Open.*

eTable 2. Quality of Included Studies per Newcastle-Ottawa Scale for Quality Assessment of Cohort Studies

Study	Selection			Comparability on basis of design and analysis	Outcome		
	Representative-ness of the exposed cohort	Selection of the non-exposed cohort	Ascertainment of exposure		Ascertainment of outcome	Adequate follow-up	Adequacy of follow-up of cohorts
Anthony, 2016	**	**	*	**	**	**	**
Boehm, 2011	**	**	*	**	**	*	**
Brummett, 2006	**	**	**	*	**	**	**
Engberg, 2014	**	**	*	**	**	**	**
Giltay, 2004	**	**	*	**	**	**	**
Giltay, 2006	**	**	*	**	**	**	**
Grossdardt, 2009	**	**	*	*	**	**	**
Hansen, 2010	**	**	*	**	**	**	**
Kim, 2011	**	**	**	**	*	*	**
Kim, 2016	**	**	**	**	**	**	**
Kubzansky, 2001	**	**	**	**	**	**	**
Mosing, 2012	**	**	**	**	**	**	**
Nabi, 2010	**	**	**	**	**	**	**
Tindle, 2009	**	**	**	**	**	**	**
Weiss-Faratci, 2017	**	**	**	**	**	**	**

Asterisks are the star ratings per the Newcastle-Ottawa Scale; * and ** indicates the highest ratings for these categories.

© 2019 Rozanski A et al. *JAMA Network Open.*

eFigure. Funnel Plots to Assess Publication Bias for (A) Cardiovascular Events and (B) All-cause Mortality

A.

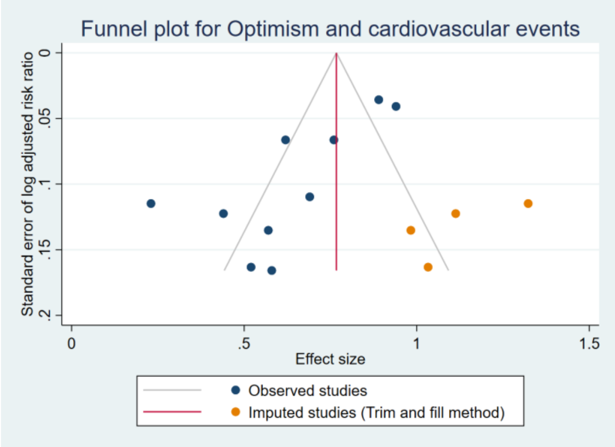

B.

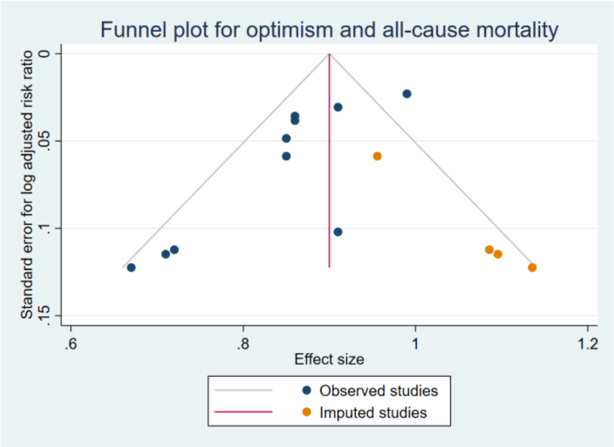

© 2019 Rozanski A et al. *JAMA Network Open.*
